# Global feather orientations changed by electric current

**DOI:** 10.1016/j.isci.2021.102671

**Published:** 2021-05-31

**Authors:** Ting-Xin Jiang, Ang Li, Chih-Min Lin, Cathleen Chiu, Jung-Hwa Cho, Brian Reid, Min Zhao, Robert H. Chow, Randall Bruce Widelitz, Cheng-Ming Chuong

**Affiliations:** 1 Department of Pathology, Keck School of Medicine, University of Southern California, 2011 Zonal Avenue, Los Ángeles, CA 90033, USA; 2 Department of Physiology and Biophysics, Keck School of Medicine, University of Southern California, Los Angeles, CA 90033, USA; 3 Department of Ophthalmology & Vision Science, and Department of Dermatology, University of California, Davis, Sacramento, CA 95816, USA

**Keywords:** biological sciences, cell biology, developmental biology

## Abstract

During chicken skin development, each feather bud exhibits its own polarity, but a population of buds organizes with a collective global orientation. We used embryonic dorsal skin, with buds aligned parallel to the rostral-caudal body axis, to explore whether exogenous electric fields affect feather polarity. Interestingly, brief exogenous current exposure prior to visible bud formation later altered bud orientations. Applying electric pulses perpendicular to the body rostral-caudal axis realigned bud growth in a collective swirl, resembling an electric field pointing toward the anode. Perturbed buds show normal molecular expression and morphogenesis except for their altered orientation. Epithelial-mesenchymal recombination demonstrates the effects of exogenous electric fields are mediated through the epithelium. Small-molecule channel inhibitor screens show Ca^2+^ channels and PI3 Kinase are involved in controlling feather bud polarity. This work reveals the importance of bioelectricity in organ development and regeneration and provides an explant culture platform for experimentation.

## Highlights

• Feather orientation is collectively altered by 3-pulsed electrical stimulations
• The electric field topology reshapes polarity cues in the epithelium
• Perturbation implicates involvement of Ca^2+^ channels, not planar cell polarity
• Molecular characterization shows feather buds are otherwise normal

## Graphical abstract

**Figure:**
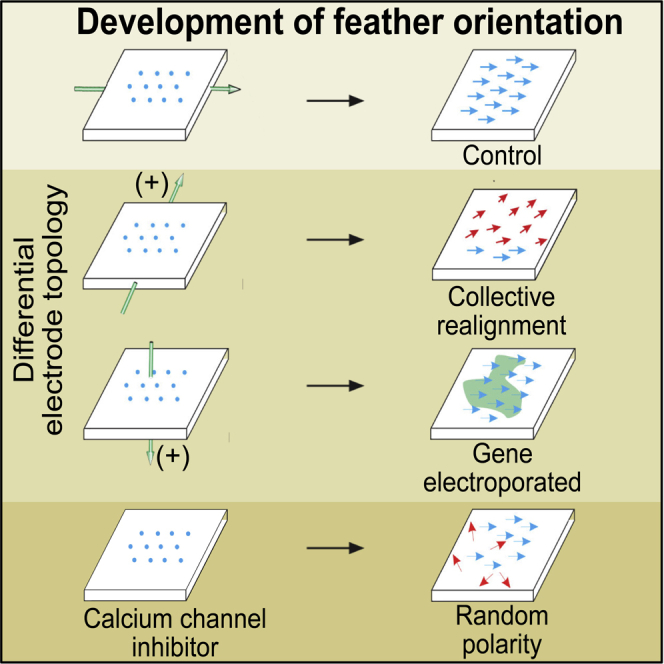

## Introduction

Collective cell migrations are known to play essential roles during processes of gastrulation, organogenesis, and wound healing. They can function in immune responses, chronic inflammation, and cancer invasion (Friedl and Gilmour, 2009; Theveneau and Mayor, 2013). Collective cell migration also can reorient tissues. Our understanding of tissue orientation in response to growth factors and molecular signals has progressed based on molecular studies performed on epithelial appendages: Drosophila appendage models (McNeill, 2010), mammalian hairs (Chang et al., 2016), and avian feathers (Chuong et al., 1996). In Drosophila, the planar cell polarity (PCP) pathway was found to regulate cuticle bristle and wing hair orientation by reorienting individual epithelial cells (reviewed in (Zecca and Struhl, 2007)). In vertebrates, homologs of PCP genes were found to be involved in orienting several tissues including hair follicles, Merkel cells, tongue papillae, and so on. (Chang et al., 2016; Wang et al., 2016). Mutations in the PCP pathway (Frizzled 6, Vangl1 + Vangl2, or Celsr1) have led to reorientation of hairs forming whorls and ridges (Cetera et al., 2017; Chang et al., 2015; Devenport and Fuchs, 2008; Guo et al., 2004; Ravni et al., 2009; Wang and Nathans, 2007; Wang et al., 2006, 2010). Interestingly, these multicellular mini-organs are composed of epidermis and dermis. Combined, these studies suggest the PCP pathway is involved in regulating skin appendage orientation in a variety of vertebrate and invertebrate species.

Bioelectricity has been shown to affect cell migration in cultured cells (Zhu et al., 2020). Most studies on bioelectricity have explored their role in cultured cells. Different cell types have been found to differentially respond to exogenous fields. Lengthy, continuous exogenous electric fields (EFs) can induce many cell types to migrate toward either the cathode or the anode (Cho et al., 2018; Hammerick et al., 2010; Hinkle et al., 1981; Iwasa et al., 2018; Jaffe and Poo, 1979; Nuccitelli and Smart, 1989; Soong et al., 1990; Yang et al., 2019). The directionality of cell migration is thought to be regulated by the electrophoresis of membrane components (Allen et al., 2013). In some cell types, this may be regulated by ERK1/2 activity as seen in bovine lens epithelia and human astrocytes (Wang et al., 2003; Yang et al., 2019). Interestingly, other cells do not change migratory behavior in response to EFs (Sillman et al., 2003). Both pulsed and focal EFs altered neurite migration (Patel and Poo, 1984) presumably by altering the endogenous EFs. The ability of electricity to regulate cell migration has been elegantly demonstrated using a programmable EF generator to control multi-axis renal epithelia and primary skin keratinocyte electrotaxis (Zajdel et al., 2020).

One interesting finding was that the motility of cell collectives can differ from that of individual cells. For example, the motility of MCF10A cell collectives was more sensitive to EF strength than individual MCF10A cells but alignments of cell collectives occurred more slowly than individual cells (Lalli and Asthagiri, 2015). At low density, cultured cancer breast cells seem to ignore EFs but migrate as a cell collective toward the anode when grown as a high density monolayer (Zhu et al., 2020). These findings suggest that endogenous currents, ion channels, and the ions they carry produce intercellular EFs that may be regulated dynamically under different cellular conditions. The effects of these signaling events can differentially regulate cell behavior.

EFs can also influence collective cell behavior. Bioelectricity has been implicated in many aspects of cell biology (Stanley and Friedman, 2019). EFs have been shown to affect tissue morphogenesis (Cervera et al., 2019; Levin, 2013) and regeneration (Levin et al., 2017). Exposing Acetabularia to a continuous light source can wake them from a state of hibernation. This induces a rhythmic polarized electrical field which leads to their growth morphogenesis (Borghi et al., 1983). In planaria, gap junction-mediated cell:cell transfer controls cellular membrane potential. Inhibiting gap junctions prior to bisection produced an inheritable new trait, the formation of 2-headed planaria (Cervera et al., 2019; Levin, 2013). EFs also regulate cell migration patterns during early stage chicken and Xenopus embryo (Funk, 2015; Hotary and Robinson, 1990; Levin and Stevenson, 2012; Ross, 2016). In Xenopus, bioelectric currents guide craniofacial patterning and tail regeneration. Producting a range of membrane potential can induce a complete eye to form from the tail region (Levin, 2013). *In vivo*, wounds can induce EFs that differentially regulate the migration of epithelial cells, dermal cells (Guo et al., 2010) and epithelial stem cells (Li et al., 2012) to promote wound healing.

The distinct morphology and arrangement of feather buds, provides a great system in which to visualize the effects of exogenous EFs. During normal development, each feather bud displays a distinct anterior-posterior (A-P) orientation. While the mechanism underlying global feather orientation is not understood, A-P polarity in the adult follicle, controlled by a Wnt 3a gradient, determines the position of the rachis, the major backbone of the feather (Yue et al., 2006). The A-P axis is also influenced by retinoic acid (Chuong et al., 1992) and Wnt 7a (Widelitz et al., 2000) gradients which lead to polarized Notch-Delta pathway expression (Chen et al., 1997). We also employed a tissue transplantation strategy used to identify polarization activity in the limb bud (Saunders, 1972) to identify a zone of “feather polarizing activity”, positioned in the posterior feather bud mesenchyme (Li et al., 2013). This zone exhibits localized Wnt 7a – nuclear beta-catenin – non-muscle myosin 2B activity that is surrounded by Notch1 expressing dermal cells. Most interestingly, inhibiting Wnt 7a, nonmuscle myosin 2B or notch signaling can randomize feather orientations (Li et al., 2013). However, randomization of feather bud orientation occurs on an individual bud basis rather than affecting the global or collective feather bud population. Using a genetic approach, bird breeders have selected for feather variants including those with altered orientations of neck feathers pointing forward as seen in Jacobin pigeons. Genetic studies showed that the ephrin pathway is involved in establishing this altered feather orientation (Shapiro et al., 2013). With many pathways showing effects on feather orientation in different ways, it is possible that the control of A-P orientation axis is a complex trait that can be controlled and modulated by multiple signaling modules, including both biochemical and bioelectric signals.

We recently explored endogenous electric currents in early stages of chicken skin morphogenesis and found that endogenous current affected collective cell migrations (Li et al., 2018). We further identified Ca^2+^ oscillations that coordinate proximal to distal mesenchymal cell movements within a feather bud, through Shh-dependent modulation of intracellular gap junctions (Li et al., 2018). EFs were able to regulate the movement of cells within cultured feather explants as well. Using optogenetics to stimulate CRAC channel activation, we demonstrated that manipulating Ca^2+^ channels could remotely modulate cell behaviors in tissues and organs close to the body surface (Li et al., 2018). These findings led us to consider that EFs may be involved as one of the signals coordinating cell behavior in building complex organ architectures.

This feather development model offers an opportunity to simultaneously analyze quantitative effects on single buds and the global orientation changes of feather bud populations. Here, embryonic feather bud orientation was explored by applying exogenous pulsed EFs. Pulsed fields have been used extensively in developmental and skin biology studies. In gastrula and neurula staged salamander embryos, pulsed field stimulation caused abnormal morphogenesis and embryonic patterning (Metcalf and Borgens, 1994). Disrupting endogenous currents with conductive implants produced developmental defects in the chicken tail, limb bud and head (Hotary and Robinson, 1992). Pulsed EFs have also been found to induce telogen to anagen transition in hair follicles of Sprague-Dawley rats (Khan et al., 2015). More recently high voltage pulsed EFs led to the restoration of scarless wound healing, including hair regeneration 6 months after treatment, possibly due to preservation of the extracellular matrix (Golberg et al., 2016). Pulsed EFs were shown to enhance the healing of diabetic wounds by altering biomechanical properties (Kwan et al., 2019). However, much remains to be learned about how EFs control tissue morphogenesis.

Our results demonstrate that feather reorientation occurs in an EF-dependent manner. It is also dependent on the axis of EF delivery. When the EF is applied perpendicular to the A-P axis, buds swirl toward the positive pole. When electrodes are aligned along the epithelial-mesenchymal axis, gene transfer can occur but feather reorientation is not observed. Epithelial-mesenchymal recombination studies show that EF effects are exerted through the epithelium. EFs alter feather bud orientation but bud morphology and molecular expression patterns appear normal. Feather bud orientation can also be randomized by exposure to Ca^2+^ channel inhibitors, suggesting that Ca^2+^ channels may be involved in preserving the memory of early exogenous EF exposure in epithelial cells. How bud orientation is determined at the single bud level and also collectively with other buds is a fascinating question explored in this study.

## Results

### Exogenous pulsed EFs induce feather buds to reorient in ovo

**Figure 1:**
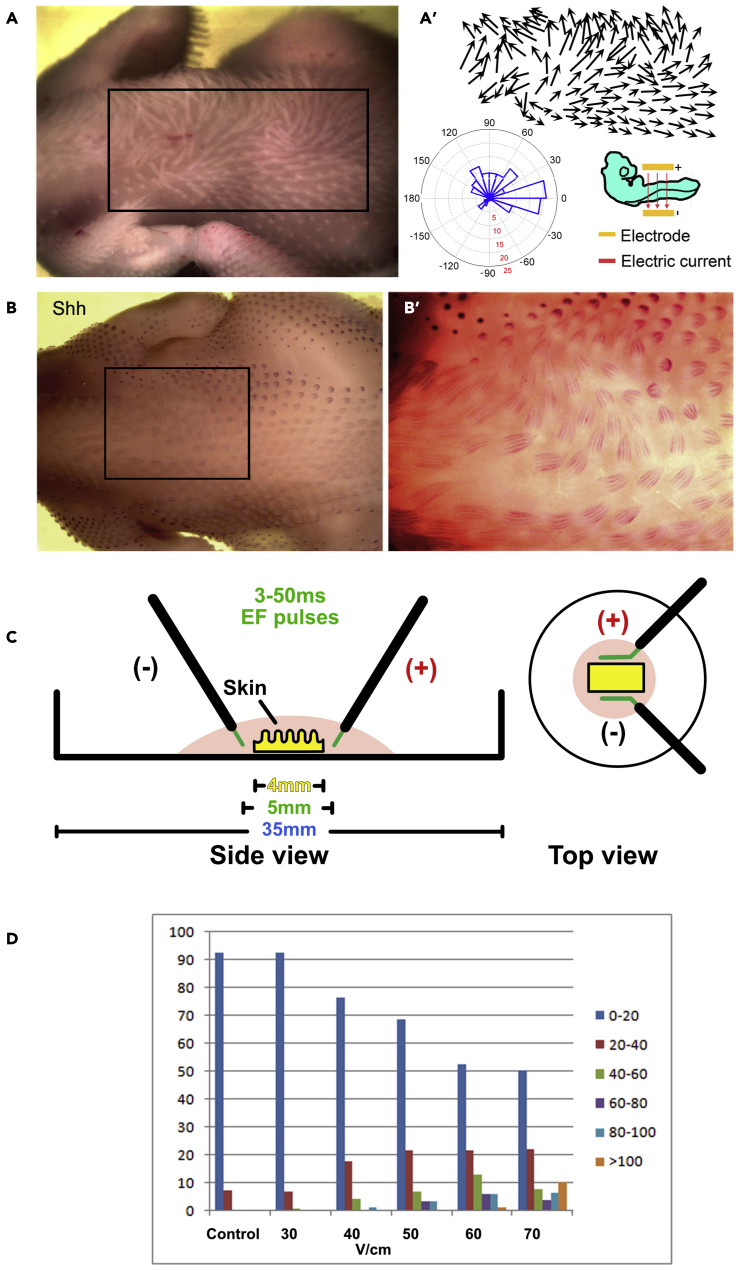
Application of exogenous electric field in ovo at E3 alters feather bud orientations collectively EF was delivered parallel to the A-P axis (anode, top; cathode, bottom). (A) Embryo exposed to EF at E3 and grown in ovo for 8 days (n = 20 × 10^3^ chicken embryos). Feather bud reorientation toward the anode is evident. A′) Feather bud orientation and length are indicated by the arrows. Feather bud orientation is also indicated by the graph. (B) Whole mount *in situ* hybridization after 7 days in ovo shows SHH RNA expressed in a normal distribution pattern within each feather bud. (B′) Higher magnification view of panel (B). (C) Schematic diagram showing side and top views of how the electric field is delivered is delivered to skin explants *in vitro* showing the placement of the electrodes relative to the explant. (D) Divergence from the original feather bud A-P axis is correlated with the voltage per centimeter applied.

To test whether an exogenous current can perturb feather bud morphogenesis, we applied three 60V, 50ms (8.46 mA) electric pulses with an electroporator (BTX ECM 830) across the medio-lateral axis of E3 (HH St. 18) skin *in ovo* (Metcalf and Borgens, 1994; Patel and Poo, 1984). After incubating 8 days in ovo, the embryos were removed and observed. A large cluster of feathers were seen to collectively reorient while ensuing outgrowth that normally occurs from the anterior (A) to posterior (P) axis veered toward the anode in a significant number of embryos (n = 20 embryos; Figure 1A). Feather length and orientation were abstracted into vectors for quantification (Figure 1A’). *In situ* hybridization of Shh (a marker of the distal feather tip) helps to visualize feather polarity divergence from the body A-P axis (Figures 1B and 1B′). We were struck by the ability of short electric pulses, administered days prior to early skin development, to alter the orientation of the skin appendages.

We next evaluated the effect of EF strength on feather reorientation. Top and side view schematic diagrams of our EF delivery system are shown (Figure 1C). Briefly, E7 skin explants were placed in 100 ul hypoosmotic solution in a 35 mm culture dish. Positive and negative electrodes were immersed in the solution on either side of the explant. E7 (HH St. 31) embryonic dorsal skin samples were exposed to three 50ms pulsed electric currents at either 30V/cm (4.23 mA), 40V/cm (5.64 mA), 50V/cm (7.05 mA), 60V/cm (8.46 mA), or 70V (9.87 mA). The samples exposed to exogenous pulsed EFs were immediately plated on Falcon culture inserts and incubated for an additional 5 days (n = 3 skins per applied EF). As the voltage increased from 30 to 70V/cm, the feathers diverge further from the original A-P axis (Figure 1D). If we assume that the endogenous voltage is 1V/cm, then the exogenous current was approximately approximately 30 to 70-fold higher, our data shows that the degree of feather bud mis-orientation positively correlated with exogenous EF strength.

We measured the effects of EF application on the temperature of the skin using a digital laser infrared thermometer. The temperature in the skin rose 0.28°C +/− 0.13°C–1.38°C +/− 0.76°C as the voltage increased from 30 to 70V/cm. It is unlikely that this change in temperature played a role in reorienting the feather buds.

### Exogenous pulsed EFs applied to *in vitro* skin explant cultures induce feather buds to reorient toward the anode

**Figure 2:**
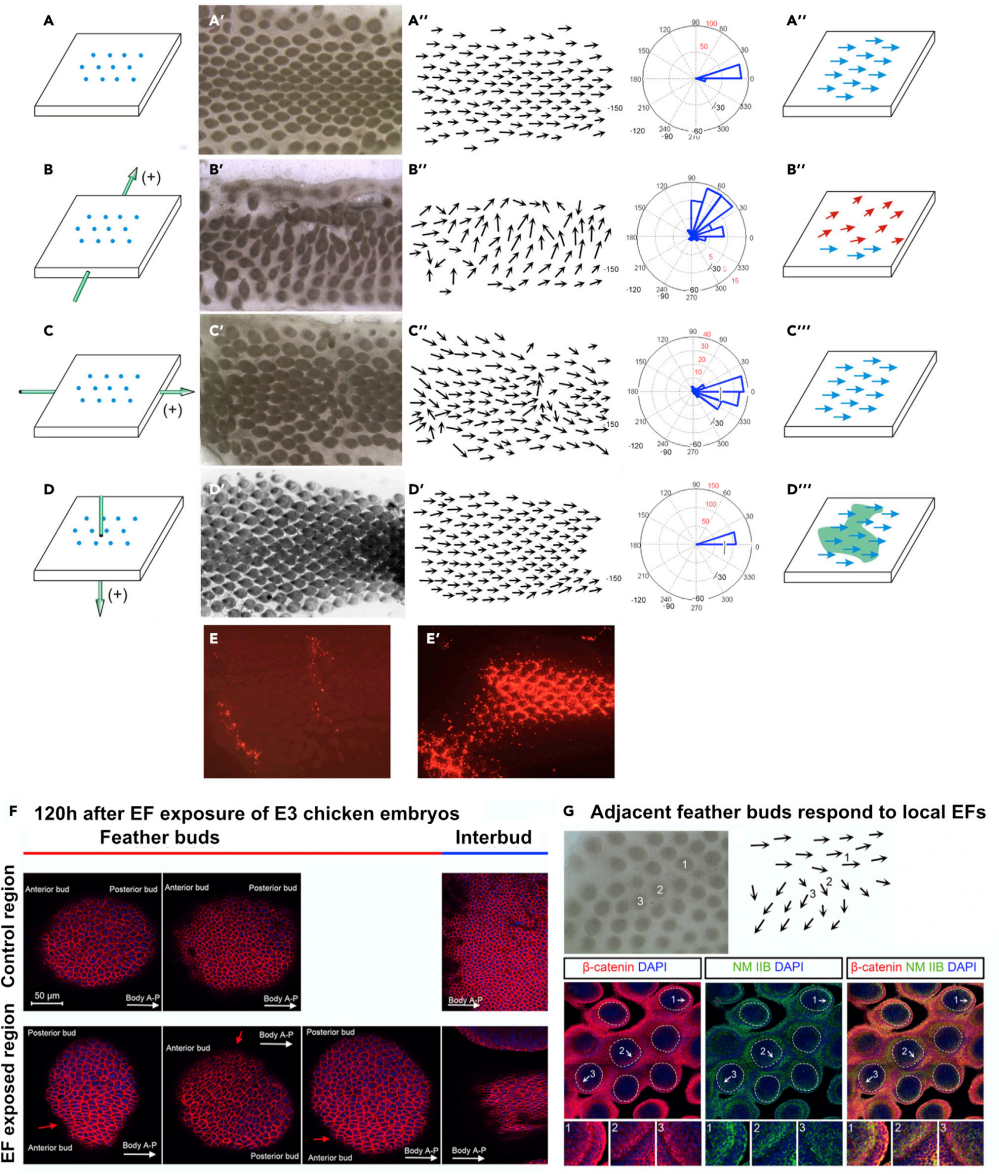
Application of exogenous electric field on E7 skin explants *in vitro* alters feather bud orientations in a topological dependent fashion (A–D) Schematic diagrams of EF flow. (A) Control skin explants with anterior (left) and posterior (right). (B) Exogenous EF exposure laterally through the skin explant with the anode (top) and cathode (bottom) poles positioned perpendicular to the A-P axis. (C) with the cathode and anode positioned along the A-P axis of the explant. (D) with the cathode above the epithelium and anode below the mesenchyme so the electrons flow from the top to the bottom of the explant perpendicularly. Feather buds (A′–D′) and their orientation (A″–D″) are shown for each condition (n = 7 skins). Gene transfer was assessed 24 hr after pulsed EF application of CMV-RFP with electrodes positioned as in panels B, C, and D. (E) Few cells expressed the exogenous plasmid when electroporated along the A-P axis. (E′) Widespread RFP expression was seen when the EF was oriented along the epithelium-mesenchyme axis. (A‴–D‴) Schematic representation of the results. (F) After transient EF exposure at E3, cell shape was assessed at E8 by staining the membranes with antibodies to E-cadherin. Cells in the EF exposed region (red arrows) within the anterior and posterior feather bud and the interbud became elongated along the axis of electroporation compared to nonelectroporated control regions. (F and G) Occasionally adjacent cells near the border of electroporated regions were subject to different EF field orientations inducing different polarity on each bud (G, top left panel). A schematic diagram shows electrode placement and EF polarity (G, top right panel). This can be seen more clearly in the polarity of beta-catenin, non-muscle myosin IIB (NM IIB) or both after immunostaining (G, bottom panels—see insets for enlargements of each bud). Size bars in panel F = 50 μM.

Next, we assessed how the orientation of the exogenous EF affects the feather orientation. For this set of experiments, we used E7 dorsal skin explant cultures so we can efficiently position the electrodes (n = 7 skins per sample). Pulsed EFs three 60V/cm, 50ms (8.46 mA) applied either perpendicular or parallel to the A-P axis. The EFs were also applied through the epithelium to mesenchyme axis. In unexposed controls, approximately 75% of feathers grew oriented along the A-P axis with ∼20% diverted by +/− 20° after 4 days in culture (Figure 2A-A‴). The minor deviated buds are at the periphery of the explants and appear to be affected by bio-mechanical force, which will be reported in a separate study. Therefore, for this study, we eliminated buds located near the edge of the skin. Applied EFs perpendicular to the A-P axis of the explant reoriented buds toward the anode (Figures 2B–2B‴): about 20% grew at an angle of +/− 40° and another 20% grew at an angle of +/− 60° from the A-P axis. A significant proportion of feather buds were diverted even further from the A-P axis. When the current was aligned with the A-P axis of the explant, feather orientations were comparable to those in controls (Figures 2C–2C‴). When electrodes were placed below the mesenchyme and above the epithelium to align the current across the explant perpendicularly (20 volts/cm 50 ms for 3 pulses), feathers were not reoriented (Figures 2D–2D‴). However an exogenous plasmid, such as CMV-RFP, was effectively transferred to the recipient skins (Figure 2E’). This contrasts with the few cells that incorporated CMB-RFP when the EF was administered along the A-P axis (Figure 2E). These results demonstrate that the polarity of exogenous electric currents applied to the skin explants exerted different effects on feather reorientation or gene transfer.

We then sought to understand how the feather buds became reoriented after exposure to the three 60V/cm, 50ms (8.46 mA) pulsed EFs. Since control feather orientation is mediated by epithelial cells (Novel 1973), we examined the keratinocyte aspect ratio within control and to skins exposed to pulsed EFs at E8 (Figure 2F). Epithelial cells in feather buds of control skin showed a normal cuboidal shape with an average aspect ratio of 1.50 (n = 463 cells). Within the interbud, the average aspect ratio of cells in control skin was 1.57 (n = 116 cells). However, following EF exposure at E3, epithelial cells became elongated along the axis of the EF and within the buds had an average aspect ratio of 2.07 (n = 443 cells) and within the interbud regions (red arrows) had an average aspect ratio of 3.93 (n = 605 cells). These data suggest that cells within the interbud regions have a greater response to the EF than those in the bud regions, possibly due to the reduced thickness of the interbud. The new anterior and posterior regions are indicated in each panel versus the body A-P axis.

We next examined the orientation of buds located near the exogenous pulsed EF (Figure 2G. We were curious as to whether buds within the EF would each respond equally to the pulsed currents. An example of this is shown in the indicated feather field. Three buds are indicated (1, 2, 3). As can be seen in the upper right, buds toward the top do not respond to the exogenous EF, as exemplified by bud 1. Buds just below that region are affected by the EF and show different degrees of reorientation. For example, Bud 2 is partially reoriented (300°) while bud 3 is shifted further (210°). Visualizing feather bud orientation is facilitated by staining for β-catenin and non-muscle myosin IIB (NM IIB). Higher magnification views of stained posterior feather bud regions for each feather bud show that although they are reoriented the posterior buds all are enriched for nuclear beta-catenin and NM IIB as was previously found in the zone of “feather polarizing activity” (Li et al., 2013). This demonstrates that the EF field produces a collective bud movement with individual buds responding to the topology of the local EF they encounter.

### Molecular characterization shows normal feather buds developed with altered orientation

**Figure 3:**
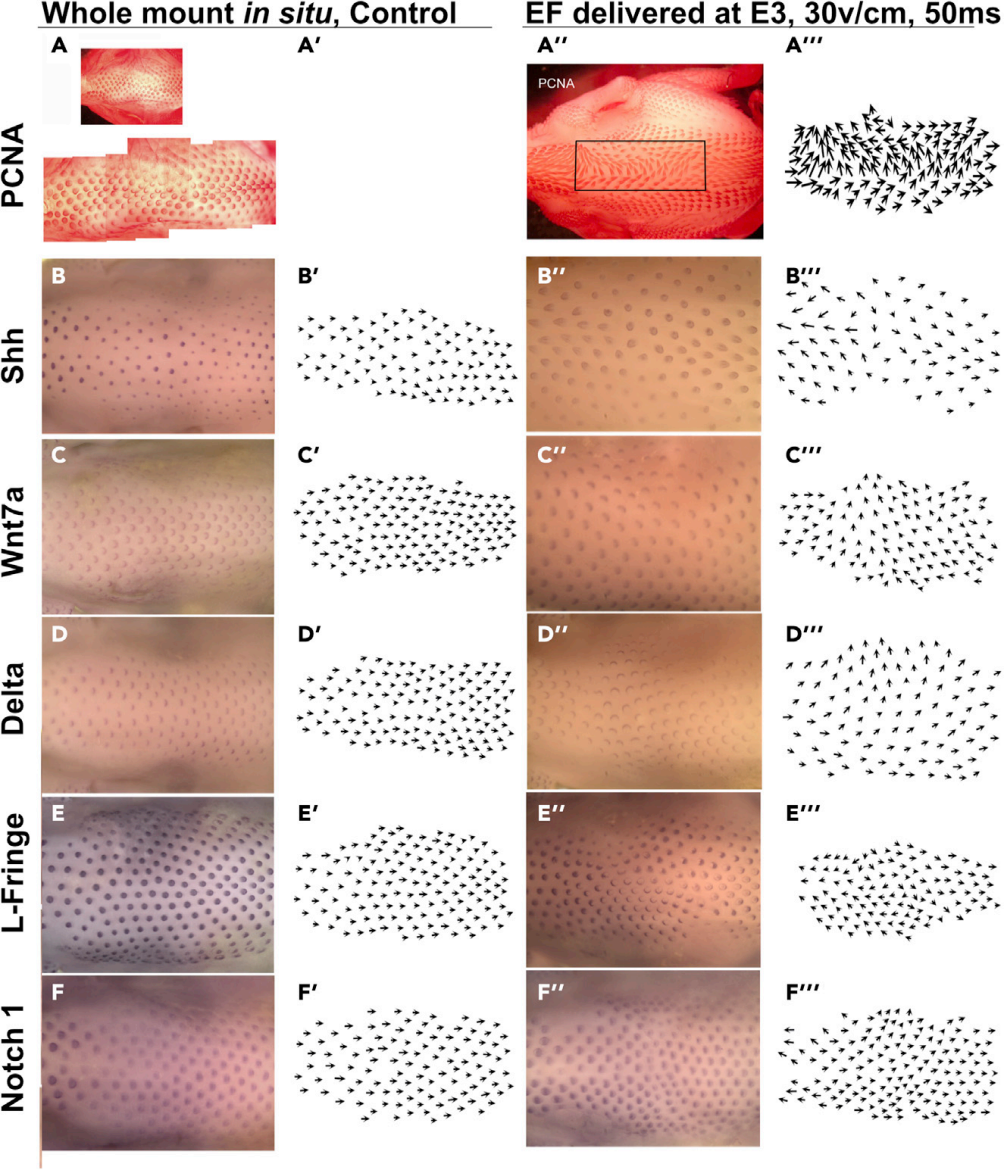
Molecular characterization of feather buds whose orientations were altered by EFs A and A″, PCNA staining to visualize proliferating cells after 6 days in culture shows each feather's localized growth zone is shifted following EF exposure. Arrows in panels A'-F' and A‴-F‴ indicate feather bud polarity. Obvious changes in feather polarity are visible after 7 days in culture (right panel). (A-F′) E3 embryos were exposed to EFs and grown until E8. Shh (B and B″), Wnt 7a (C and C″), Delta 1 (D and D″), L-Fringe (E and E″) and Notch1 (F and F″) mRNA expression patterns at E8 were detected by *in situ* hybridization. The relative position of each gene is unchanged in EF exposed skin, but the buds are reoriented. n = 5 embryos per sample.

We examined the impact of extrinsic pulsed three 60V/cm, 50ms (8.46 mA) electrical current on subsequent proliferation and downstream molecular expression during feather development (Figure 3). We exposed E3 chicken embryos to exogenous EF pulses (anode top) and assessed the expression of PCNA (A and A″), Shh (B and B″), Delta 1 (C and C″), Wnt 7a (D and D″), L-Fringe (E and E″) and Notch1 (F and F″) at E8 (n = 5 skins for each molecule). Whole mount PCNA immunostaining shows feather bud localized growth zone (LoGZ) activity 7 days after exposure to the exogenous pulsed EF. Proliferation is restricted to posterior regions of control feather buds but becomes shifted toward the anode after EF exposure. The reoriented buds develop normally as indicated by proper sonic hedgehog (Shh) mRNA localization in the marginal plates. Gene expression within the developing buds was similar in normally oriented and reoriented feathers. Therefore, the expression patterns within the regions exposed to EFs shifted their alignment to coincide with the new feather bud orientation, but the development of individual feather buds appear to be normal. This suggests that the genes analyzed passively respond to rather than actively contribute to feather bud reorientation.

### Exogenous electrical current acts on the epithelium to reorient feather buds

**Figure 4:**
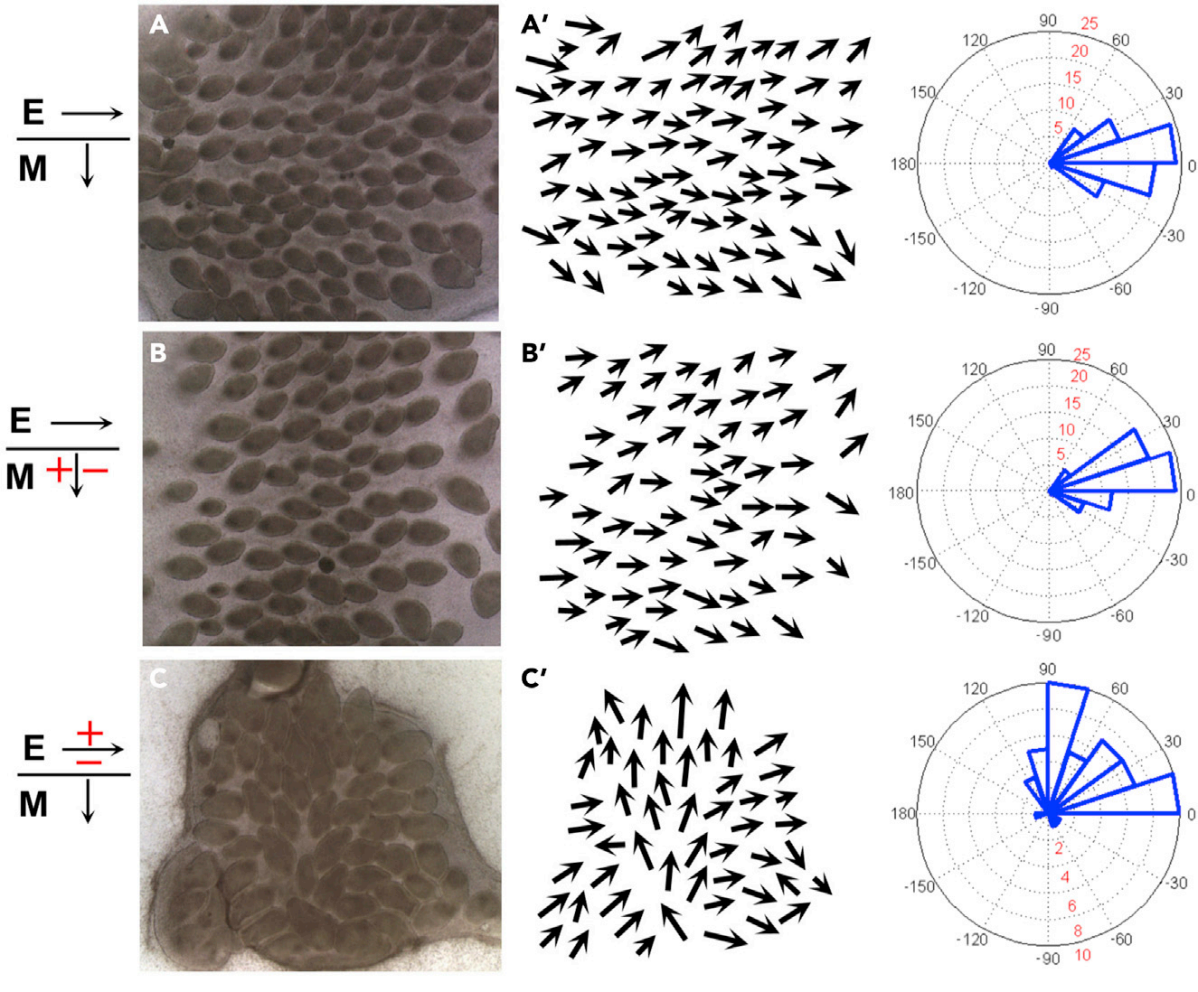
Tissue recombination experiments show exogenous EF induced feather bud reorientation through acting on the epidermis (A–C) Electrode positioning (red +/−) and initial epithelial and mesenchymal orientation (black arrows) are shown (n = 5 explants per sample). (A′) Control, epithelium recombined with control mesenchyme. Feather polarity was dictated by epithelial orientation. (B′) Control epithelium recombined with EF exposed mesenchyme (cathode on the left). Feather buds still grew with epithelial orientation. (C′) EF exposed epithelium (cathode to the top) recombined with control mesenchyme. Most feather buds turned toward the cathode (A′–C′, arrows).

Feather bud polarization is known to be initiated by epithelial signals during normal skin development (Novel, 1973). To distinguish whether pulsed EFs reorient feather buds by affecting epithelial or mesenchymal signals, we independently subjected the epithelium or mesenchyme to EFs. This was done by soaking the skin in 2x calcium and magnesium free solution and peeling the epithelium from the mesenchyme. Exogenous EFs (three 60V/cm, 50ms) were applied to the epithelium (6.87 mA) or mesenchyme (11.02 mA). Afterward the epithelium was rotated 90° relative to the mesenchyme and placed back on the mesenchyme to form a recombined skin (Jiang et al., 1999) (Figure 4; n = 5 skins per sample). The effect on feather reorientation was then measured. For each experiment, epithelial and mesenchymal orientation is indicated by the black arrows and EF polarity is indicated by the red +/−. Mesenchyme exposed to EFs perpendicular to the A-P axis before recombination with control epithelium had little effect on feather polarity. Exposing the epithelium to EFs aligned perpendicular to the A-P axis before recombination with control mesenchyme caused most feather buds to reorient toward the site where the anode was originally placed. These data demonstrate that electric current can reorient feather buds by acting specifically on the epithelium.

### Planar cell polarity molecules are expressed in feather buds but do not appear to be the cause of reorientation

**Figure 5:**
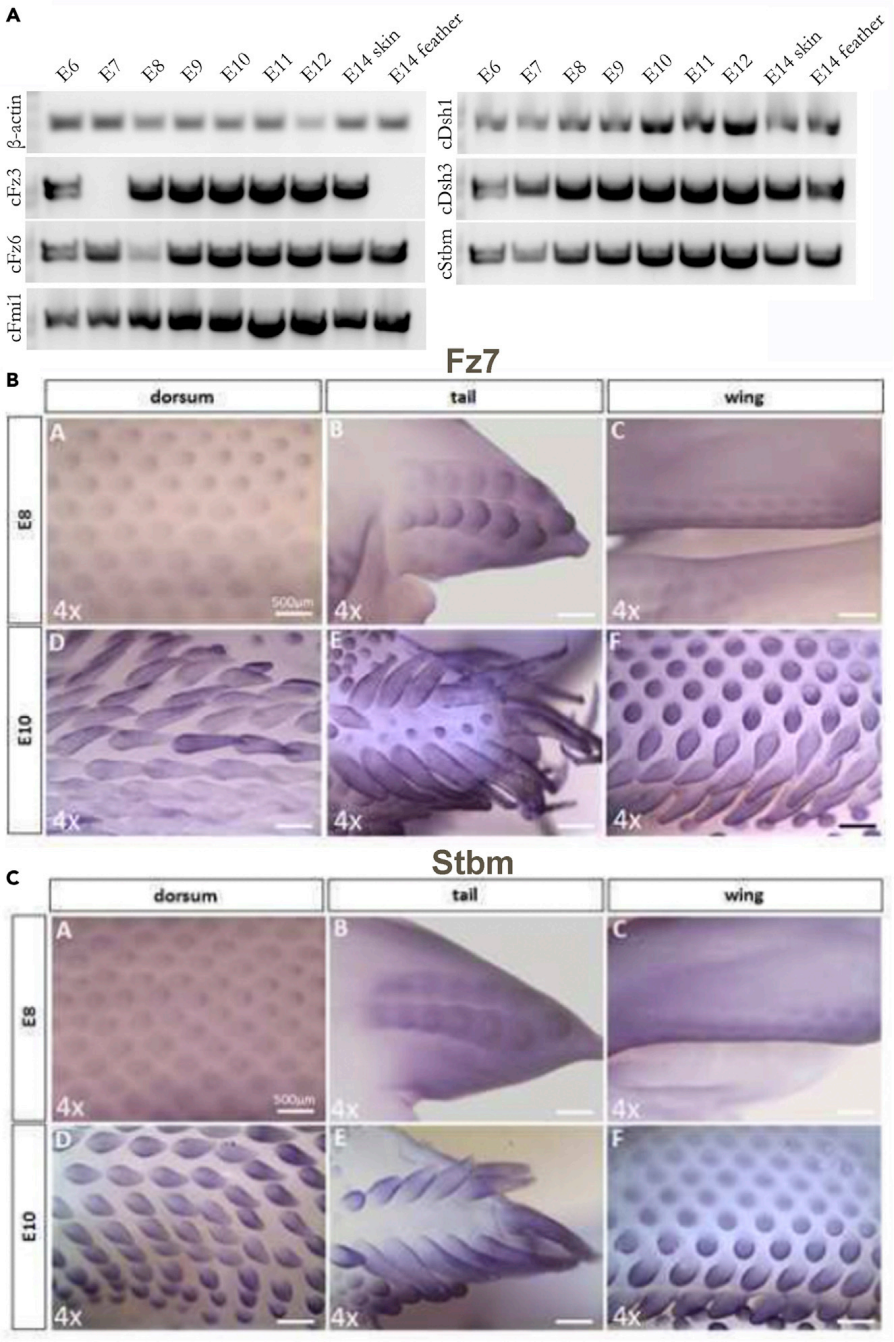
Evaluating the roles of planar cell polarity genes in exogenous EF induced feather bud reorientation (A) Semi-quantitative PCR verified the presence PCP genes Fz3, Fz6, Fmi1, Dsh1, Dsh3, and Stbm in cDNAs amplified from E6-E14 dorsal skin. (B and C) Whole mount *in situ* hybridization of normal E8 and E10 chicken skin using probes against Fz7 and Stbm. In the E8 dorsal tract, Fz7 is expressed as a ring in the bud epithelium and asymmetrically localizes to the posterior epithelium at the short bud stage. A similar pattern is shown in the tail and the wing tracts. Once the feather bud elongates at E10, Fz7 is expressed in the epithelium from the feather base to the distal tip in the dorsal, tail and wing tracts. Fz7 is absent from the interbud regions. At E8, Stbm initially is expressed as a spot and ring-like pattern and progresses to asymmetrically localize to the posterior epithelium in the dorsal tract. Asymmetry is not observed in the tail and wing tracts. Once the feather bud elongates at E10, Stbm is expressed in the epithelium but becomes absent in the proximal region (n = 3 samples per time point). Size bars in panels C and D = 500 μm.

To examine the possible role of PCP in mediating feather bud reorientation in response to exogenous EFs, we examined expression kinetics of the PCP molecules cFz3, cFz6, cFmi1, cDsh1, cDsh3 and cStbm in skin at E6, E7, E8, E9, E10, E11, E12 and E14 using PCR amplification (n = 3 skins per time point). Each gene began to be expressed at E6. Expression levels gradually elevated until approximately E11 and then decreased (Figure 5A). We then characterized Strabismus (Stbm) and Frizzled 7 (Fz7) expression using *in situ* hybridization. Stbm is first expressed throughout feather buds and progressively localizes to an asymmetric expression pattern predominantly in the posterior feather buds at E8 (Figure 5C). At E10, expression is found in the epithelium but is absent from the proximal feather bud region. Fz7 shows a similar expression pattern as Stbm at E8 and becomes distributed throughout the feather bud epithelium at E10 (Figure 5B). Since PCP molecules do not appear with polarized expression in the skin until times well after the exogenous EF exposure, they are unlikely to be involved in mediating feather bud orientation in this scenario. This will be verified with a more in-depth study in future experiments.

### Calcium channels are involved in EF mediated feather bud reorientation

**Figure 6:**
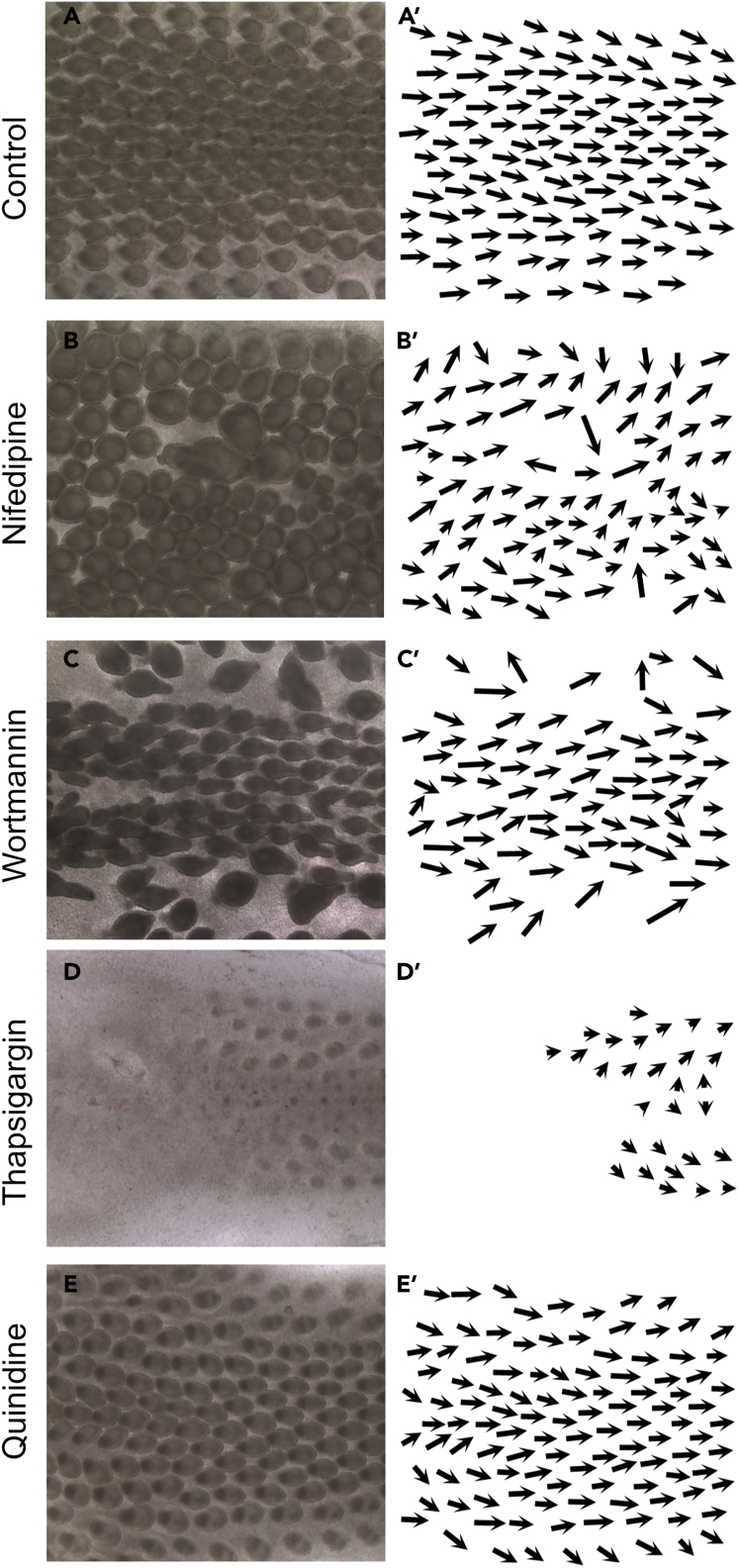
Evaluating the roles of ion channel inhibitors on EF induced reorientation of feather buds (A-F) Examples of specimens treated with inhibitors. (A) Control untreated sample. Specimens treated with (B) Nifedipine, (C) Wortmannin, (D) Thapsigargin, or (E) Quinidine. (A'-E') Feather orientations are indicated by the arrows.

We were struck by the fact that although embryonic skin does not begin to form until E6.5, brief EF pulses at E3 could lead to changes in feather polarity. We wondered how information about feather polarity was stored within the tissue. Our results demonstrate that known feather polarity molecular cues and planar cell polarity molecules do not play a role in this process. This suggested that the information might be stored as differences in membrane potential mediated by ion channels may regulate feather axis development in feathers. EFs are thought to regulate several biological events. Endogenous currents likely modulate membrane potential through voltage sensitive H^+^, Ca^2+^, K^+^ and Na^+^ channels to modulate cell behaviors (Levin, 2007). To begin to dissect the role of these pathways in our system, we previously ran RNA-seq transcriptome analyses examining ion channels whose expression was changed between E7 and E11 (Li et al., 2018). We found expression levels of genes encoding K^+^ channels, Kcnab1, Kcnk17 and a Ca^2+^ channel component, Cacng3, were elevated at E9 compared to E7, whereas Kcnk5 and Cacna1h were elevated at E7. So, we tested several ion channel inhibitors on skin explants (Table 1; Figure 6; n = 3 skins per inhibitor). Thapsigargin is an inhibitor of the ER Ca^2+^ pump that elevates cytosolic Ca^2+^ levels (Jones and Sharpe, 1994). Here, 1 μM Thapsigargin suppressed feather bud elongation. Only Nifedipine, an L-type Ca^2+^ channel blocker, and Wortmannin, a PI3 Kinase inhibitor caused feathers to grow in random directions, indicating that Ca^2+^ channels may be involved in the biological mechanism interpreting exogenous EFs. Our lab earlier showed that 50 μM Nifedipine could reduce the KCl-induced Ca^2+^ response in skin explants and block feather buds from developing polarity (Li et al., 2018). Here, we waited an extra day and saw that polarity was randomized. The specific channels and how they are involved will be investigated further.

**Table 1:** List of channel inhibitors

A. Inhibitor	Known mode of action	Phenotype
BAPTA	Ca^2+^ chelator	Normal
Cadmium	Block voltage-dependent Ca^2+^ channels	Induces dermal condensation (1 mM from
	from fluxing calcium ions	beads), thickens dermis and inhibits
		feather bud formation (5 uM in media)
Fendiline	Voltage gated Ca^2+^ channel inhibitor	Inhibits feather bud formation (100 uM)
Verapamil	L-type Ca^2+^ channel blocker	Induces feather buds under beads (10 uM),
		inhibits feather formation (100 uM)
Gadolinium chloride trihydrate	Ca^2+^ channel blocker	Normal
Diltiazem	L-type Ca^2+^ channel blocker	Inhibits feather bud growth (100 uM)
Thapsigargin	Raises cytoplasmic Ca^2+^ levels	Inhibits feather bud growth (1 uM)
Quinidine	Potassium channel blocker	Normal
Ameloride HCL	Selective T-type Ca^2+^ channel blocker and	Induces buds around the beads (10 uM),
	blocker of epithelial Na^+^ channel	inhibits medial feather buds (50 um)
		while lateral buds are enlarged
Lidocaine	Na^+^ channel blocker	Normal
Flecainide	Na^+^ channel blocker	Inhibits feather bud growth (80 uM)
Ouabain	Selective Na^+^, K^+^-ATPase inhibitor	Inhibition of feather buds (25 uM)
Tetraethylammonium chloride	Non-selective K^+^ channel blocker	Inhibition of feather buds (50 uM)
Tetrodotoxin	Selective inhibitor of Na^+^ channel conductance	Normal
Nifedipine	L-type Ca^2+^ channel blocker	Alters feather bud orientation (50 uM)
Silver Nitrate	Increases Cl^−^ efflux and sodium influx	Inhibits feather bud formation (250uM)
Furosemide	Inhibits chloride efflux	Normal
5-(N-Ethyl-N-Isopropyl) Amiloride	Selective T-type Ca^2+^ channel blocker and	Feather buds become thinner (10 uM)
	blocker of epithelial sodium channel	and inhibited (25 uM)
Wortmannin	PI3 Kinase inhibitor	Alters orientation of younger buds
		and causes bud fusion (25 uM)

## Discussion

During tissue morphogenesis, cells migrate through their 3-dimensional environment and become organized and patterned into appropriate configurations (Li et al., 2015). EFs have been shown to lead to tissue polarization. This is easily seen in the formation of heads and tails in planaria. When gap junction activity is suppressed regenerating planaria develop 2 heads, one at either end (Levin, 2013). Similarly Xenopus treated with sodium ionophore can be induced to regenerate their amputated limbs with complete polarity including the distal digits (Levin, 2013). Left-right asymmetry in Xenopus embryos is established by the Nodal, Lefty Pitx2 axis but the epigenetic silencing of genes on the left side is mediated by the electrotaxis of morphogens (Carneiro et al., 2011). Hence, growth factors, signaling molecules and adhesion molecules regulate cell behaviors to achieve complex tissue patterns. Biochemical signals may not be sufficient to drive the whole morphogenetic process. Integration with mechanical force and bioelectric signals have been implicated in regulating tissue morphogenesis (McLaughlin and Levin, 2018; Muncie and Weaver, 2018; Silver et al., 2020). To dissect out the morphogenetic control of tissue patterning, we chose a model consisting of a population of mini-organs. Mini-organ systems include Drosophila larva bristles (Cho et al., 2020), omatitidia formation (Jenny, 2010), mammalian hairs (Wang and Nathans, 2007) and avian feathers (Chen et al., 2015). The unique aspect of the “*mini-organ population model*” is that it is made of repetitive mini-organ units that facilitate the analysis of factors that control each mini-organ unit or factors that affect the global arrangement of mini-organs.

Using a vibrating probe, we recently detected unexpected dynamic changes in endogenous bioelectric currents in developing chicken dorsal skin. The currents originally flow into the whole skin and early feather bud. Yet, at the time feather buds are elongating, a small outward electric current is transiently measured in the anterior region of each bud, from HH stage 35 to stage 36. This suggests the whole dorsal skin EF is split into numerous mini-EFs, corresponding to each elongating feather bud (Li et al., 2018). We started to examine the significance of these bioelectric currents at the single feather bud level and global population level.

### Single feather bud level

We were able to visualize calcium oscillations using GCaMP6s in feather bud mesenchymal cells. Before the initiation of elongation, Ca^2+^ signals occurred sporadically and randomly. When buds started to elongate, calcium oscillations became synchronized and cells started to move distally in a collective manner. This process requires epithelia Shh and Wnt signals, and also connexin-43 dependent gap junction communications. Perturbation of the above process leads to disoriented feather buds. Thus we demonstrate that the coupling of biochemical signals with bioelectric signals, mediated by Ca^2+^ channels and gap-junctions, can coordinate mesenchymal cell migration. These function together with other mechanisms to organize a single feather bud with the correct polarity (Li et al., 2018).

### Global feather bud population level

Disrupting a tissue's polarizing mechanism might be expected to randomize feather polarity. However, when we use reagents (e.g., nifedipine, carbenoxolone, PMA and mefloquine that perturb calcium channel activities, cells moved upwards but did not align along the A-P axis (Li et al., 2018). Yet, here we found that electric pulses applied prior to feather placode formation elicited a coordinated, collective response that appeared days later (a placode has ∼250 um diameter containing ∼300 epithelial cells and ∼800 mesenchymal cells). The affected feathers become aligned in swirl patterns resembling a wind vector map, potentially reflect the topology of the EF. Since the EF response occurs at an early developmental stage, we surmise that the exogenous electric current might alter endogenous ion channel activities as was shown in other systems. Electric pulses induced Ca^2+^ fluctuations in mesenchymal stem cells (Hanna et al., 2017) and calcium gated BK K^+^ channels in glioblastoma cells (Burke et al., 2017). We first examined ion channel expression during feather bud development through transcriptome analysis at E7 and E9 when polarity changes begin to be observed (Li et al., 2020). These include Kcnab1, Kcnk 5, Kcnk17, Cagng3 and Cacna1h, which show different temporal and spatial expression. We then tested the role of ion channels (Ca^2+^, Cl^−^, H^+^, K^+^, Na^+^, etc) with specific inhibitors to evaluate their involvement in establishing feather polarity. While some inhibitors had general effects on feather morphogenesis, only Ca^2+^ channel inhibitors specifically affected cell polarity. Since mutations in the PCP pathway (Frizzled 6, Vangl1 + Vangl2, or Celsr1) were found to reorient hairs into whorled patterns (Cetera et al., 2017; Chang et al., 2015; Devenport and Fuchs, 2008; Guo et al., 2004; Ravni et al., 2009; Wang and Nathans, 2007; Wang et al., 2006, 2010), we also explored the timing of PCP gene expression during the EF induced changes in the feather model and found that PCP genes are expressed later in feather development, after feather buds were reoriented, suggesting that the control of global orientation precedes PCP rearrangement.

How might calcium channels affect feather bud alignment collectively? EF polarity is critical to global changes within feather buds, since the posterior end of feathers grow toward the anode. A polarized sub-cellular distribution of Ca^2+^ channels and cytoskeleton changes, brought about by high localized calcium concentrations, could potentially reorient feathers. In planaria, Ca^2+^ channels regulate polarized muscle regeneration (Chan et al., 2017). In Xenopus, the H^+^- K^+^ ATPase help to establish polarized left-right asymmetry (Levin et al., 2002). In mice, the KCNJ13 K^+^ channel is essential to establish proper tracheal smooth muscle polarity (Yin et al., 2018). In chicken feathers, mesenchymal Ca^2+^ signaling through voltage-gated Ca^2+^ channels and/or Ca^2+^ release activated channels are coordinated through Shh and Wnt induced formation of gap-junction networks (Li et al., 2018). On a practical note about the importance of the topology of electrodes during exposure to the exogenous pulsed EFs, when molecules are transported across cell membranes by applying the electric current perpendicular to the explant from the epithelium (cathode) to the mesenchyme (anode), there is little shift in feather orientation, but extracellular plasmids are transduced effectively by the EF application.

To summarize features of EFs' role in orienting a population of feather buds: First, manipulating endogenous EFs by supplying short term exposure to pulsed exogenous EFs at E3 (HH stage 18), several days before feather buds form (at E6-7, HH stage 29-31), can later alter global feather bud polarity within the affected field during morphogenesis. This suggests that the integument has mechanisms to remember and to respond to EF stimulation. Second, the reorientation event is not random, but makes a collective swirl pattern dependent on the direction of the exogenous EF. Third, organ reorientation is mediated by the epithelium not the mesenchyme. Fourth, organ morphogenesis occurs normally in the reoriented feather buds. Fifth, perturbation of organ orientation can be uncoupled from gene transfer produced by exposure to exogenous EFs, depending on the topological positions of cathodes and anodes, a note to be considered for those using EF application for gene transfer to study their role in epithelial cell biology. Sixth, this represents a collective feather response to changes in their local environment.

Therefore, feather orientation appears to involve (1) local factors within a single multicellular feather bud that determines A-P polarity, and (2) global factors that pattern collective feather bud orientation, noting that relative orientation can change between adjacent buds. We recently identified ion channels involved in coordinating cell movements within a single feather bud that can lead to feather polarity determination (Li et al., 2018).

### Limitations of study

At the global level, we must acknowledge we have not determined how these activities are coordinated and how a bud senses the axial orientation of its neighbors. One possibility is through the re-arrangement of channel proteins. Yet channels usually exist in very small amounts, and we were not able to identify sub-cellular distributions of the channels with immunostaining. It also has been suggested that mini-injury within sub-cellular organelle re-organization may contribute to tissue reorientation. We have not been able to definitively establish the role of bioelectricity; however, we did carry out tissue interaction level experiments to demonstrate the effect is mediated via the epidermis. While we will continue to explore molecular interactions that occur between cells of single feather buds and those in the global population, we decided to publish this fascinating finding which we think provides new insights to the morphogenesis field beyond the chicken skin model. We expect these findings will inspire new studies into bioelectricity as an important factor guiding tissue morphogenesis in terms of embryo development, wound regeneration, and stem cell engineering.

## STAR★Methods

### Key resources table

**Table:** 

REAGENT OR RESOURCE	SOURCE	IDENTIFIER
**Chemicals, peptides and recombinant proteins**		

5-(N-Ethyl-N-Isopropyl) Amiloride	Tocris	Compound CID: 1795; Cat. No. 3378
BAPTA	Tocris	Compound CID: 104751; Cat. No. 2786
Cadmium	Sigma Aldrich	Compound CID: 23973: Cat. No. GF58541272
Diltiazem	Tocris	Compound CID: 39186; Cat. No. 0685
Fendiline	Tocris	Compound CID: 3336; Cat. No. 6407
Flecainide	Tocris	Compound CID: 3356; Cat. No. 1470
Furosemide	Tocris	Compound CID: 3440; Cat. No. 3109
Gadolinium chloride hexahydrate	Sigma Aldrich	Compound CID: 71308319; Cat. No. G7532
Lidocaine	Tocris	Compound CID: 3676; Cat. No. 3057
Nifedipine	Tocris	Compound CID: 4485; Cat. No. 1075
Ouabain	Tocris	Compound CID: 439501; Cat. No. 1076
Quinidine	Tocris	Compound CID: 441074; Cat. No. 4108
Silver Nitrate	Sigma Aldrich	Compound CID: 24470; Cat. No. 209139
Tetraethylammonium chloride	Tocris	Compound CID: 5946; Cat. No. 3068
Tetrodotoxin	Tocris	Compound CID: 5946; Cat. No. 1078
Thapsigargin	Tocris	Compound CID: 446378; Cat. No. 1138
Wortmannin	Tocris	Compound CID: 312145; Cat. No. 1232

**Experimental models: organisms**		

Chicken embryos: Egg, SPF, Premium	Charles River	Cat. No. 10100326

**Software and algorithms**		

MatLab	MathWorks	RRID:SCR_001622; https://www.mathworks.com/products/matlab.html

### Resource availability

#### Lead contact

• Further information and requests for resources and reagents should be directed to and will be fulfilled by the lead contact Cheng-Ming Chuong (cmchuong@usc.edu).

#### Materials availability

• This study did not generate new unique reagents.

#### Data and code availability

• This study did not generate/analyze datasets or code

### Experimental model and subject details

• White Leghorn Chicken embryos – Specific pathogen-free (SPAFAS) White Leghorn chick embryos were obtained from Charles River Laboratories (Franklin, CT) and incubated at 37.5°C. Embryos were staged according to Hamburger and Hamilton (Hamburger and Hamilton, 1951).
• Skin explants obtained from White Leghorn Chicken embryos – Explant cultures and skin recombination were performed in the manner described previously (Ting-Berreth and Chuong, 1996). Briefly, dorsal embryonic chicken skin was dissected at embryonic day 6.5 and cultured on Falcon culture insert membranes at the air:DMEM media containing 10% fetal bovine serum interface.
• Epithelial-mesenchymal recombination studies – E6 – E8 chicken skin was submerged in 2x calcium, magnesium free solution at 4°C and then the epithelium was gently peeled from the mesenchyme using fine forceps. The mesenchyme was then rinsed and plated on Falcon culture insert membranes and overlaid with the epithelium. The orientation of the epithelium relative to the mesenchyme is indicated.

### Method details

• ***In vivo* electric field application** – Whole White Leghorn chicken embryo EF application was performed at E3 (H&H stage 18). The egg was windowed and a portion of the chorio-allantoic membrane removed to expose the embryo. The electrodes were placed closely adjacent to the two sides of the embryo’s body. The electric current was delivered from a BTX Electro Square Porator ECM 830 through genetrodes (BTX model 512 with an angled 5mm gold tip) for 3 pulses at 50 ms in at the indicated field strength. Electrode spacing was 4mm. After treatment, the egg was sealed with scotch tape, and incubated for another 6 to 8 days at 37°C.
• ***In vitro* electric field application** – Intact skin explants (E7) or recombined skin explants measuring ∼4mm in width were placed in a 35 mm culture dish and covered with 100 ul of hypoosmotic buffer (Eppendorf). Electrodes separated by 5 mm were placed as indicated in hypoosmotic buffer (Figure 1C). EFs delivered from a BTX Electro Square Porator ECM 830 through genetrodes (BTX model 512) for 3 pulses at 50 ms at the indicated field strength. These explants were then plated on culture inserts (Falcon) and allowed to grow at 37°C as described (Jiang et al., 1998). To visualize gene transfer efficiency in these studies. CMV-GFP was introduced to the hypoosmotic buffer prior to the application of the EFs. The explants were then incubated for 4 days and visualized by fluorescence microscopy.
• **Measurements of current –** Since current is not reported by the BTX ECM 830, we measured the resistance of the skin and hypoosmotic solution and used the formula V=IR to determine the current applied (mA).
• **Calculation of electric field** strength – Electric field strength (V/cm) was determined per sample using the voltage applied divided by the distance between the probes (0.5 cm).
• **Measurement of temperature** – We also measured the effects of EF application on the temperature of the skin using a digital laser infrared thermometer. The temperature in the skin rose 0.28°C +/- 0.13°C to 1.38°C +/- 0.76°C as the voltage increased from 30 to 70V/cm (n = 6 skin explants per voltage).
• ***In situ* hybridization and immunohistochemistry** Skin and embryos were fixed in 4% paraformaldehyde and processed for RNA whole mount *in situ* hybridization or immunohistochemistry (Jiang and Chuong, 1992; Ting-Berreth and Chuong, 1996). An antisense RNA probe was prepared for *in situ* hybridization. Antibodies against PCNA (Sigma) were used for immunohistochemistry.

### Quantification and statistical analysis

• **Feather length and orientation measurements** - Photographs of the skin, skin explants and recombined skin explants were enlarged to measure feather lengths and orientations. Length information was tabulated in Microsoft Excel and the average +/- standard deviation was calculated. Orientation data was entered into MatLab and analyzed using the Polar Plot function. In the text and Figure Legends we have stated whether n = the number of embryos/explants or the number of feather buds.
• **Cell aspect ratio measurements** – Using higher magnification images, we were able to determine the maximum length versus width of individual cells. We then calculated the cell aspect ratio in control or EF treated samples. For these data we report n as the number of cells used for these determinations.
